# Novel antimony-based antimicrobial drug targets membranes of Gram-positive and Gram-negative bacterial pathogens

**DOI:** 10.1128/spectrum.04234-23

**Published:** 2024-04-23

**Authors:** Tarosha Salpadoru, Kevin E. Pinks, Jacob A. Lieberman, Kaitlyn Cotton, Karen L. Wozniak, Nikolay Gerasimchuk, Marianna A. Patrauchan

**Affiliations:** 1 Department of Microbiology and Molecular Genetics, Oklahoma State University, Stillwater, Oklahoma, USA; 2 Department of Chemistry and Biochemistry, Missouri State University, Springfield, Missouri, USA; JMI Laboratories, North Liberty, Iowa, USA

**Keywords:** broad spectrum, combination therapy, cytotoxicity

## Abstract

Antimicrobial resistance (AMR) poses a significant worldwide public health crisis that continues to threaten our ability to successfully treat bacterial infections. With the decline in effectiveness of conventional antimicrobial therapies and the lack of new antibiotic pipelines, there is a renewed interest in exploring the potential of metal-based antimicrobial compounds. Antimony-based compounds with a long history of use in medicine have re-emerged as potential antimicrobial agents. We previously synthesized a series of novel organoantimony(V) compounds complexed with cyanoximates with a strong potential of antimicrobial activity against several AMR bacterial and fungal pathogens. Here, five selected compounds were studied for their antibacterial efficacy against three important bacterial pathogens: *Pseudomonas aeruginosa*, *Escherichia coli*, and *Staphylococcus aureus*. Among five tested compounds, SbPh_4_ACO showed antimicrobial activity against all three bacterial strains with the MIC of 50–100 µg/mL. The minimum bactericidal concentration/MIC values were less than or equal to 4 indicating that the effects of SbPh_4_ACO are bactericidal. Moreover, ultra-thin electron microscopy revealed that SbPh_4_ACO treatment caused membrane disruption in all three strains, which was further validated by increased membrane permeability. We also showed that SbPh_4_ACO acted synergistically with the antibiotics, polymyxin B and cefoxitin used to treat AMR strains of *P. aeruginosa* and *S. aureus*, respectively, and that at synergistic MIC concentration 12.5 µg/mL, its cytotoxicity against the cell lines, Hela, McCoy, and A549 dropped below the threshold. Overall, the results highlight the antimicrobial potential of novel antimony-based compound, SbPh_4_ACO, and its use as a potentiator of other antibiotics against both Gram-positive and Gram-negative bacterial pathogens.

**IMPORTANCE:**

Antibiotic resistance presents a critical global public health crisis that threatens our ability to combat bacterial infections. In light of the declining efficacy of traditional antibiotics, the use of alternative solutions, such as metal-based antimicrobial compounds, has gained renewed interest. Based on the previously synthesized innovative organoantimony(V) compounds, we selected and further characterized the antibacterial efficacy of five of them against three important Gram-positive and Gram-negative bacterial pathogens. Among these compounds, SbPh_4_ACO showed broad-spectrum bactericidal activity, with membrane-disrupting effects against all three pathogens. Furthermore, we revealed the synergistic potential of SbPh_4_ACO when combined with antibiotics, such as cefoxitin, at concentrations that exert no cytotoxic effects tested on three mammalian cell lines. This study offers the first report on the mechanisms of action of novel antimony-based antimicrobial and presents the therapeutic potential of SbPh_4_ACO in combating both Gram-positive and Gram-negative bacterial pathogens while enhancing the efficacy of existing antibiotics.

## INTRODUCTION

Since their discovery, antibiotics have become an essential part of the modern healthcare, allowing treatment of otherwise fatal infections (1–3). Years of improper use of these compounds lead to antimicrobial resistance (AMR) in bacteria posing a significant and urgent threat to public health on a global scale (4–6). According to a global systematic analysis conducted in 2019, there were an estimated 4.95 million annual fatalities associated with bacterial AMR (6). This death toll was estimated to reach 10 million by 2050 emphasizing the pressing need for novel antimicrobials that are effective and safe (7–9). Despite the urgency, the current pipeline of developing new antibiotics remains insufficient to counter the rising resistance in bacterial pathogens (10, 11). Only 12 new antimicrobials have been approved since 2017, and 10 of them belong to already existing classes, which undermines their effectiveness due to the risk of rapid resistance development (11, 12). With the increasing demand for novel antimicrobial strategies, metals and metal-containing compounds have gained renewed interest as potential antimicrobials (13–16). One of the advantages of using metal-based antimicrobials is the observed capacity to target multiple cellular processes in bacteria leading to pleiotropic effects, a property that decelerates the evolution of resistance (14, 17). Furthermore, the ease with which the physicochemical properties of metal-based complexes can be fine-tuned by incorporating a variety of organic ligands makes them more attractive (13, 18).

The two metals, antimony (Sb) and bismuth (Bi), have a long history of use in medicinal chemistry, which dates back to the 18th century (19). Sb and its salts have been used by ancient Egyptians and Assyrians to treat a variety of ailments including fevers and skin irritation (18, 20, 21). Since early 20th century, Sb-based drugs have been used to treat leishmaniasis, an infective parasitic disease caused by the protozoan *Leishmania* (22). In addition to their antiparasitic activity, several organoantimony compounds have also been studied for their potential as antimicrobial, antifungal, antiviral, and anticancer agents (18, 23–27). Previously, we synthesized and characterized a series of novel organoantimony(V) cyanoximates. These compounds, denoted by the general formula Sb(C_6_H_5_)_4_L, with L representing the ligand, were obtained by subjecting AgL (or TlL) and Sb(C_6_H_5_)_4_Br in CH_3_CN to high-yield heterogeneous metathesis at room temperature (28). The eight cyanoximate ligands used in our previous study belong to a new subclass of small organic molecules that are chemically and thermally stable (29) and non-cytotoxic (30–32) and exhibit a range of biological activities (33–38). When tested against a panel of drug-resistant bacterial and fungal species, including Gram-negative *Escherichia coli* and *Pseudomonas aeruginosa*, Gram-positive *Staphylococcus aureus*, and fungal pathogens *Cryptococcus neoformans* and *Candida albicans*, these compounds varied in their antibacterial activity and showed promise as both broad- and narrow-spectrum antimicrobials (28, 39).

Here, we studied in more detail the antibacterial efficacy of selected five organoantimony cyanoximates and investigated the underlying mechanism of action for SbPh_4_ACO against three pathogens *P. aeruginosa*, *E. coli*, and *S. aureus*.

## RESULTS AND DISCUSSION

### The tetraphenyl organoantimony cyanoximate, SbPh_4_ACO, inhibits the growth of pathogenic strains of *P. aeruginosa*, *E. coli,* and *S. aureus*

**Fig 1:**
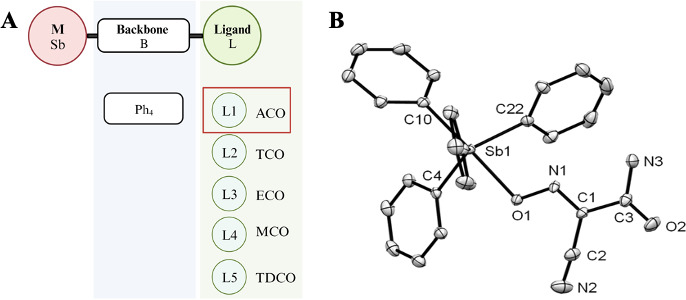
The structure of organoantimony tetraphenyl cyanoximate compounds. (A) The compounds comprise of five ligands (indicated in the green box), attached to a tetraphenyl backbone (indicated in the blue box) and an antimony (created in BioRender). The focus of this study, SbPh_4_ACO, is highlighted in red. Created in BioRender.com. (B) Molecular structure of Sb(Ph)_4_(ACO) with numbering of principal atoms only and H-atoms being omitted for clarity. Thermal ellipsoids for atoms are drawn at their 50% probability level. The compound crystallized as a clear colorless block-like specimen (Fig. S2). The cyanoxime moiety adopts the trans-anti conformation with respect to the position of the N-O of the oxime and pyridyl ring along the C2-C3 bond. The ACO-oxime anion is planar, signifying no dihedral angle, and agrees with previously mentioned literature data for this ligand. This crystal structure is stabilized by H-bonding between one of amide’s hydrogens and the cyanoximes N from N-O-Sb (Fig. S3). The geometry of the coordination polyhedron of the Sb(V) atom was a distorted trigonal bipyramid. The cyano group of the oxime is linear with atoms C1-C2-N2 = 175.9° (Table S2). Bond distances for the cyano group C2-N2 = 1.144 Å, oxime group N1-O1 = 1.337 Å, and C1-N1 = 1.278 Å. This crystal structure has been deposited at the Cambridge Crystallographic Data Centre under number 2011862 and is available upon request.

Our previous study describes the synthesis and characterization of five novel tetraphenyl organoantimony cyanoximates, namely, SbPh_4_ACO, SbPh_4_ECO, SbPh_4_MCO, SbPh_4_TCO, and SbPh_4_TDCO (Fig. 1A). These compounds share a tetraphenyl backbone with Sb attached to it but differ in their associated cyanoximate ligand groups (Fig. 1A). To evaluate their antibacterial activity, we determined the minimum inhibitory concentration of these compounds and their respective ligand controls H(ACO), H(ECO), and H(MCO) by standard methods. The ligand controls of these compounds did not show any antibacterial activity when tested at the concentration ranging from 0 to 200 µg/mL (Table 1). In contrast, three out of five Sb-based compounds showed antibacterial activity against at least two of the three pathogens tested. Among them, SbPh_4_(ACO) inhibited the growth of all three bacterial pathogens, *P. aeruginosa*, *E. coli*, and *S. aureus* with MIC values ranging from 50 to 100 µg/mL, implying a strong potential as a broad-spectrum antimicrobial. SbPh_4_ECO and SbPh_4_MCO only inhibited the growth of *P. aeruginosa* and *S. aureus*.

**TABLE 1:** Minimum inhibitory concentrations of Sb-based antimicrobials and ligand controls^*a*^

Antimicrobial compound/MIC, µg/mL	*E. coli*	*P. aeruginosa*	*S. aureus*
Ligand controls			
H(ACO)	>200	>200	>200
H(ECO)	>200	>200	>200
H(MCO)	>200	>200	>200
Sb-based antimicrobial compounds			
SbPh_4_ACO	**100**	**100**	**50**
SbPh_4_ECO	>200	**150**	**150**
SbPh_4_MCO	>200	**200**	**100**
SbPh_4_TCO	>200	>200	>200
SbPh_4_TDCO	>200	>200	>200

^*a*^ The reported numbers represent the average of three experiments. Each antimicrobial was subjected to standard CLSI broth dilution assays at concentrations 0–200 µg/mL. >200 indicates that the MIC for the given compound was not detectable at the range of concentrations tested. MICs are highlighted in bold.

The two compounds SbPh_4_TCO and SbPh_4_TDCO did not show any antibacterial activity toward any of the tested pathogens. This lack of antibacterial activity of SbPh_4_TDCO differs from our previous results that were based on disc diffusion assays and where the compound showed a growth inhibitory effect against *S. aureus* (28). It is possible that the poor solubility of the drug in liquid medium prevents its activity. This can be circumvented by structural modifications in future studies.

It is also noteworthy that the growth of *E. coli* was inhibited by only one [SbPh_4_(ACO)] out of five compounds, suggesting that this pathogen may harbor mechanisms different to those in the other two pathogens to deter the activity of these compounds. It has been reported that although *P. aeruginosa* exhibits reduced outer membrane permeability compared with *E. coli* (approximately 8% of the *E. coli* threshold), the size exclusion cut-off for its outer membrane porins is approximately 3,000 Da , markedly higher than that of *E. coli*, which is around 500 Da (40–42). This difference may contribute to the observed differences in the susceptibility to Sb-based antimicrobials between these two organisms.

To develop a more mechanistic understanding of the antibacterial activity of these compounds, we selected SbPh_4_ACO that showed growth inhibition against all three tested bacterial strains.

### SbPh_4_ACO exerts bactericidal effect on *P. aeruginosa*, *E. coli*, and *S. aureus*

**Fig 2:**
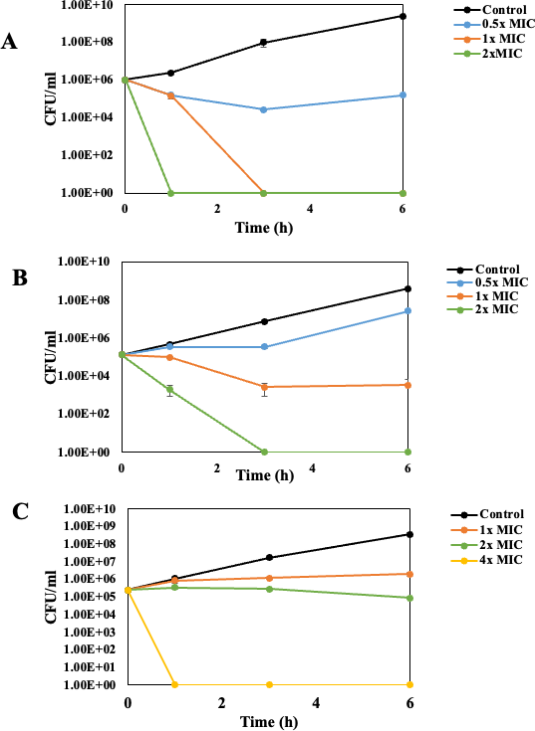
Time-kill assay of SbPh_4_ACO on *P. aeruginosa* PAO1 (**A**), *E. coli* S17 (**B**), and *S. aureus* NRS70 (**C**). Each bacterial culture was normalized to a final concentration of 5 × 10^5^ CFU/mL and exposed to different concentrations of SbPh_4_ACO; 0.5× (blue), 1× (orange), 2× (green), and 4× (yellow) of their respective MIC values. The solvent control for each strain is indicated in black. The cultures were incubated at 37°C shaking at 200 rpm for 6 h, and aliquots collected at different timepoints were used to determine the colony count. CFU/mL values represent an average of three replicates with standard error of mean (SEM). Each experiment was repeated at least once for reproducibility.

To determine whether SbPh_4_ACO has a bactericidal effect, time-kill assays were performed for three bacterial strains *P. aeruginosa* PAO1, *E. coli* S17, and *S. aureus* NRS70. The strains were grown in cation-adjusted Mueller-Hinton broth (CA-MHB), normalized to reach a standard inoculum size of 5 × 10^5^ CFU/mL as established (43), and treated with different concentrations of SbPh_4_ACO ranging from 0.5×–4× of their respective MIC values. Following the treatment, bacterial survival was monitored for 6 h by counting CFU and compared with the untreated growth control (Fig. 2).

Exposure to SbPh_4_ACO at its MIC concentration of 100 µg/mL led to 99.9% killing of *P. aeruginosa* after 3 h of incubation (Fig. 2A). For *E. coli*, the applied amount of the compounds had to be 2× MIC (200 µg/mL) in order to achieve 100% killing that also occurred after 3 h of incubation (Fig. 2B). In the case of *S. aureus*, exposure to 4× MIC (200 µg/mL) of the compound for 1 h (Fig. 2C) also led to 100% killing. So, overall, 100–200 µg/mL SbPh_4_ACO resulted in at least 99.9% reduction in 5 × 10^5^ CFU/mL, thus demonstrating its bactericidal activity against the three bacterial strains (Fig. 2).

For each bacterial strain, the MBC was determined as the concentration of the antimicrobial that eliminates 90% of the bacterial population within 6 h upon exposure which is equivalent to 99% killing at 24 h as established (44, 45). According to our observations, the MBC of SbPh_4_ACO for *P. aeruginosa*, *E. coli*, and *S. aureus* are 100, 200 and 200 µg/mL, respectively (Table 2). At these concentrations, we observed 100% killing for the three bacterial strains upon 1–3 h of exposure to this compound. Further, according to published studies (46–48), a drug is considered to have bactericidal activity when its MBC/MIC ratio is ≤4 and bacteriostatic, when the MBC/MIC ratio ≥ 8. The respective MBC/MIC ratios we obtained for SbPh_4_ACO for *P. aeruginosa*, *E. coli*, and *S. aureus* are 0.5, 1, and 4 (Table 2), further confirming the bactericidal effect of SbPh_4_ACO on these pathogens.

**TABLE 2:** MBC of SbPh_4_ACO against *P. aeruginosa* PAO1, *E. coli* S17, and *S. aureus* NRS70^*a*^

Bacterial strain	MBC (µg/mL)	MIC (µg/mL)	MBC/MIC	Interpretation
*P. aeruginosa*	100	100	0.5	Bactericidal
*E. coli*	100	100	1	Bactericidal
Methicillin-resistant *S. aureus* (MRSA)	200	50	4	Bactericidal

^*a*^ The MIC and MBC values represent the average of at least three replicates. MBC/MIC ratios ≤ 4 were considered as bactericidal.

It is generally regarded that bactericidal antimicrobials are more desirable than bacteriostatic drugs, as they prevent the regrowth of the bacteria (49). Hence, from a clinical viewpoint, the bactericidal properties of SbPh_4_ACO make it more suitable for applications to treat bacterial infections.

### SbPh_4_ACO disrupts *P. aeruginosa*, *E. coli*, and *S. aureus* membranes

**Fig 3:**
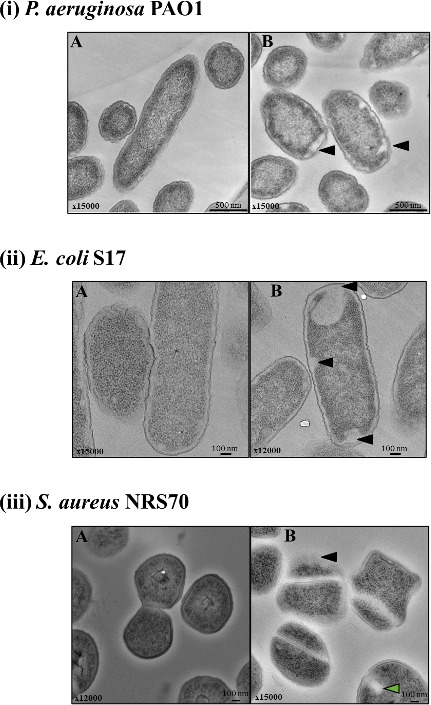
Ultra-structural changes in *P. aeruginosa* PAO1 (i)*, E. coli* S17 (ii)*,* and *S. aureus* NRS70 (iii) upon treatment with SbPh_4_ACO. Bacterial cultures were treated with SbPh_4_ACO at their MIC concentration for 1 h, pelleted, and subjected to thin-layer TEM imaging. In the DMSO-treated control (panel A) after 1 h, cells have uniform cytoplasmic density and intact cell membranes. After being treated with 1xMIC of SbPh_4_ACO (panel B) for 1 h, cells showed signs of membrane detaching from the cytoplasm. Aberrant membrane disruptions visible upon treatment are indicated by black arrows. In *S. aureus*, the apparent stalling of cell division is indicated by a blue arrow. The level of magnification and the scale bar are represented on the bottom of each image. At least four fields were examined per sample for each condition.

To determine the nature of SbPh_4_ACO impact on bacterial cells, we employed thin-layer TEM imaging. The three bacterial strains *P. aeruginosa* PAO1, *E. coli* S17, and *S. aureus* NRS70 were grown in CA-MHB for 5 h and exposed to the MIC level of the compound for 1 h. Following the treatment, their cell morphology was visualized by TEM. As a control, cells were treated with the appropriate volume of dimethyl sulfoxide (DMSO) used to dissolve SbPh_4_ACO (Fig. 3). The controls for the three strains did not show any visible signs of cellular damage. In contrast, SbPh_4_ACO-treated cells showed prominent membrane disruptions in all three species. Both *P. aeruginosa* and *E. coli* cells showed electron-light regions near the membrane indicating the separation of the cytoplasm from the membrane (shown with arrows in Fig. 3A and B). We also observed electron-light regions appearing within the cytoplasm. In *S. aureus*, in addition to apparent membrane damage, a majority of cells appeared to have stalled during the formation or separation of the septum indicating interrupted cell division (Fig. 3C). These observations suggest that SbPh_4_ACO targets the bacterial cell membrane, causing membrane disruption, with accompanying changes in cell morphology likely leading to lethal effects on bacteria.

### SbPh_4_ACO causes bacterial membrane permeabilization

**Fig 4:**
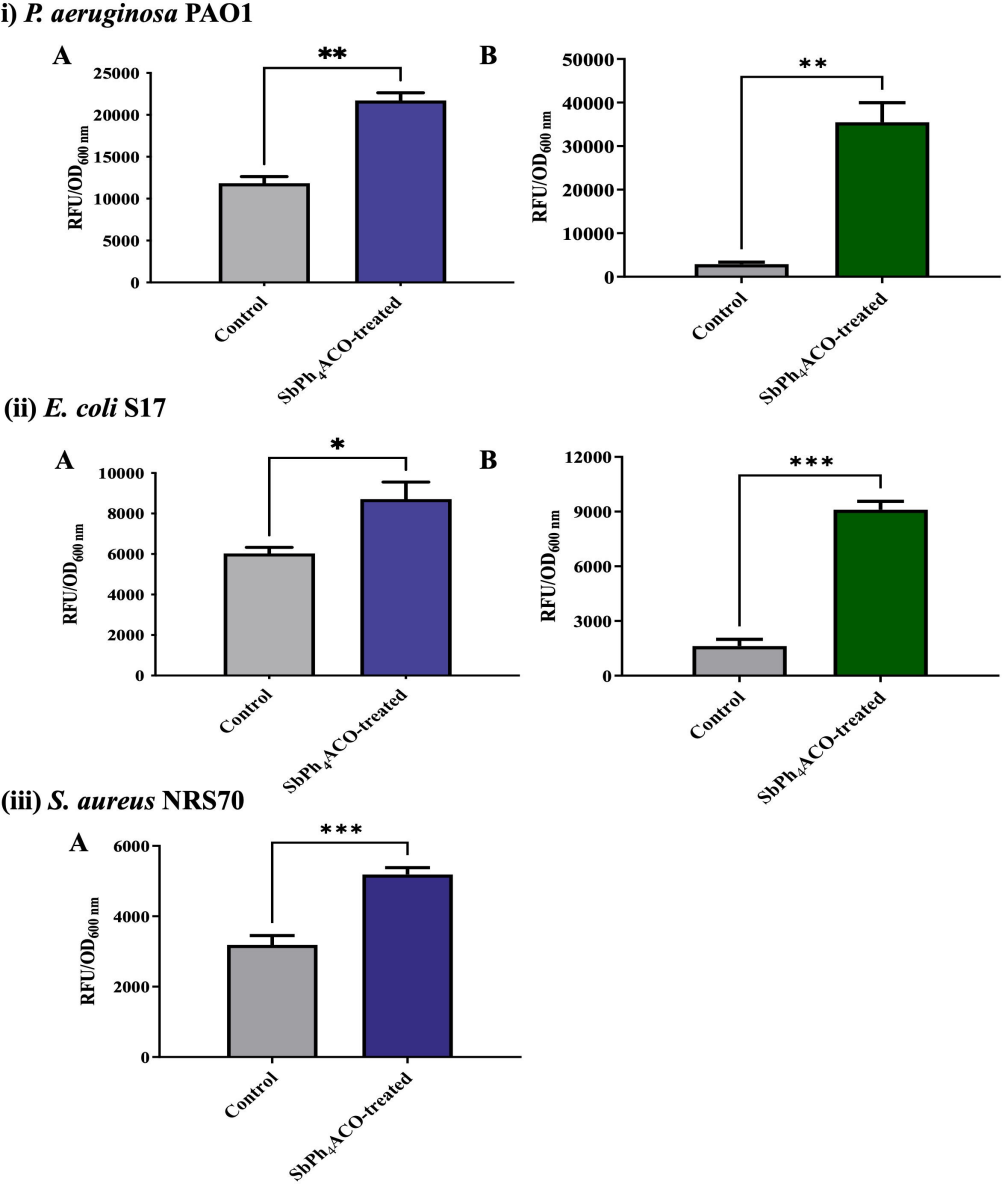
The inner (**A**) and outer (**B**) membrane permeability of *P. aeruginosa* (i), *E. coli* (ii), and *S. aureus* (iii) treated with DMSO (gray, control) and SbPh_4_ACO (colored) determined by PI and NPN uptake assays, respectively. Bacterial cultures grown for 12 h were inoculated at 1:100 into main cultures in MHB. These cultures were treated with 0.5× MIC concentration of SbPh_4_ACO for 1 h and were harvested and subjected to permeability assays using PI and NPN to determine the inner (shown in blue) and outer (shown in green) membrane permeability. The fluorescence levels of PI and NPN were measured at 535/617 nm and 350/420 nm, respectively, and were normalized by optical density at 600 nm (OD_600nm_). The values represent an average of at least three replicates along with the SEM. *P value ≤ 0.05, **P value ≤ 0.01, and ***P value ≤ 0.001.

Since the TEM results indicated that SbPh_4_ACO targets bacterial membranes, we tested its impact on membrane permeability by using the fluorescent stains NPN and PI (50–53) as probes for outer and inner membranes, respectively. For this, all three strains were exposed to SbPh_4_ACO for 1 h at the sub-inhibitory levels (0.5× MIC) and assayed for membrane permeability. In agreement with the TEM, the inner membrane permeability showed a consistent but modest increase of 1.4–1.8-fold (*P* ≤ 0.01) vs the corresponding solvent control (Fig. 4B, D and E). However, the permeability of the outer membranes of Gram-negative *P. aeruginosa* and *E. coli* increased much greater, by 12.2 and 5.6-fold (*P* ≤ 0.01), respectively (Fig. 4A, B, C and D). It is important to acknowledge that *P. aeruginosa* possess multiple efflux systems that may impact the membrane permeability, and therefore, all the permeability measurements were compared with the corresponding solvent controls. SbPh_4_ACO itself may be translocated by efflux or impact efflux of other compounds, which warrants future studies for insights into its mode of action and its potential synergy with other antimicrobials.

Our results indicate that the effect of SbPh_4_ACO on the permeability of outer membranes of Gram-negative *P. aeruginosa* and *E. coli* is more pronounced than that on the inner membranes. These differences may be attributed to lower accessibility of the inner membrane or the differences in the composition between the inner and outer membranes. For example, lipopolysaccharides in the outer leaflet of the outer membrane may be more susceptible to SbPh_4_ACO than the phospholipid bilayer that makes up the inner membrane. The outer membrane in Gram-negative bacteria is well known as a permeability barrier, particularly against hydrophobic antibiotics, detergents, and dyes (54, 55) as well as large hydrophilic drugs (42, 56–59). As a result, most Gram-negative bacteria display intrinsic resistance to several antibiotics such as oxacillin, rifampicin, erythromycin, and vancomycin (55, 60). The composition of the outer membrane is also known to undergo a variety of modifications to provide resistance to a wide range of antibiotics including β-lactams, quinolones, and colistin (61). Therefore, the ability of SbPh_4_ACO to disrupt the outer membrane and overcome this barrier can be used to potentiate the efficacy of antibiotics that are otherwise blocked by this barrier. This may expand the available therapeutic options for infections caused by Gram-negative bacterial pathogens and assist in lowering the required doses of antibiotics thereby reducing their toxicity and lowering the risk of resistance development.

### SbPh_4_ACO shows synergism with clinically used antibiotics

To explore the potential of SbPh_4_ACO to potentiate the efficacy of antibiotics and to reduce the effective dose of the compound itself, we performed a series of checkerboard assays conducted with SbPh_4_ACO in combination with polymyxin-B, meropenem and cefoxitin [an alternative to methicillin (62), used in clinic against *P. aeruginosa*, *E. coli*, and methicillin-susceptible *S. aureus*, respectively (63–66)].

Synergy is commonly determined by calculating the fractional inhibitory concentration index (FIC_I_) (67–69). FIC_I_ ≤ 0.5 indicates synergistic relationships between drugs, whereas FIC_I_ in the range 0.5–4.0 indicates an additive or indifferent relationship, and FIC_I_ > 4.0 is indicative of antagonism between the drugs (70). The FIC_I_ value calculated for the combination SbPh_4_ACO and polymyxin-B against *P. aeruginosa* reached 0.396 ± 0.055 indicating that SbPh_4_ACO acts synergistically with polymyxin-B (Table 3). Similarly, a synergistic behavior was observed for the combination of SbPh_4_ACO and cefoxitin against *S. aureus* with the FIC index of 0.5. However, the interaction between SbPh_4_ACO and meropenem resulted in the FIC_I_ of 1, which was indicative of additive/indifferent relationships between these two drugs.

**TABLE 3:** FIC index of various antimicrobial agents in combination with SbPh_4_ACO against *P. aeruginosa* PAO1, *E. coli* S17, and *S. aureus* NRS70 as determined by broth dilution and checkerboard assays^*a*^

Organism	Antibiotic	Concentration (µg/mL)	FIC index	Interpretation	MIC in the presence of antibiotic (µg/mL)
*P. aeruginosa* PAO1	Polymyxin B	0.5–32	0.4 ± 0.06	Synergistic	34.4 ± 9.4
*E. coli* S17	Meropenem	0–0.25	0.9 ± 0.1	Indifferent	50.0 ± 0
*S. aureus* NRS70	Cefoxitin	0–16	0.5 ± 0	Synergistic	17.1 ± 4.7

^*a*^ FIC index and the MIC concentration of SbPh_4_ACO when used in combination with antibiotics are represented as an average of at least three replicates with the standard error.

The strong synergy observed between SbPh_4_ACO and polymyxin-B suggests that the compounds, although may affect related processes, do not share specific targets. Given that polymyxin-B disrupts the outer membrane by targeting the LPS (71, 72) and since SbPh_4_ACO also disrupts the outer membrane and increases its permeability, it is possible that SbPh_4_ACO may not target LPS specifically. Polymyxin B was shown to synergize with different types of antibiotics, including meropenem (63, 73, 74) targeting penicillin-binding proteins (PBP) (65); however, SbPh_4_ACO showed indifferent FIC for the latter. This suggests that SbPh_4_ACO does not target PBPs. The synergistic impact of SbPh_4_ACO with cefoxitin (an alternative to methicillin) for *S. aureus*, also targeting PBPs involved in cell wall synthesis (75–77), further supports that SbPh_4_ACO may damage the outer layers of the cell wall and provide a better access to but not targeting PBPs located in the cytoplasmic membrane (78). The results indicate that SbPh_4_ACO may help salvage cefoxitin as a treatment for MRSA and reduce the dosage and therefore the toxicity of polymyxin B. Further studies are needed to discover the specific mechanisms of action and to identify antibiotics that can be used in combination with SbPh_4_ACO to treat *E. coli*.

### Compound SbPh_4_(ACO) is non-cytotoxic to mammalian cells at the synergistic MIC concentration

To determine the cytotoxic effects of SbPh_4_ACO on mammalian cells, we performed cytotoxicity assays with SbPh_4_ACO concentrations ranging from 12.5 to 200 μg/mL on three mammalian cell lines, HeLa, McCoy, and A549. These concentrations were selected based on the previously determined MIC concentrations of SbPh_4_ACO when used alone or in combination with other antibiotics (Tables 2 and 3).

**Fig 5:**
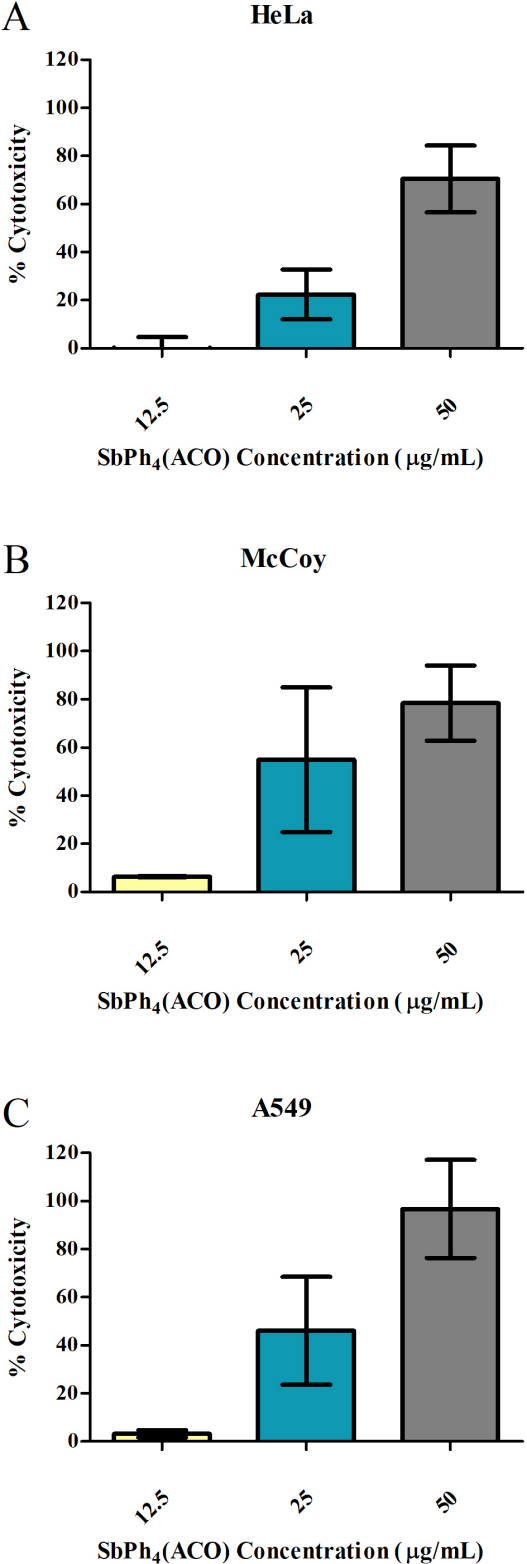
Cytotoxicity of SbPh_4_ACO compound toward three mammalian cell lines. Cytotoxicity was performed using HeLa (**A**), McCoy (**B**), and A549 (**C**) cells incubated (24 h, 37°C, 5% CO_2_) in cell culture media along with SbPh_4_ACO at MIC concentrations identified in combination with polymyxin-B and cefoxitin as determined by the checkerboard assay (12.5 µg/mL, 25 µg/mL, and 50 µg/mL, shown in yellow, blue, and gray, respectively). Percent cytotoxicity was determined per the manufacturer’s instructions. Each experiment was conducted in triplicate wells, and the data shown are the means ± SEM of the two independent experiments for each cell line. The compound exhibited low cytotoxicity (<30%) at the 12.5 µg/mL concentration in all cell lines.

When used at 12.5 µg/mL, SbPh_4_ACO exhibited minimal cytotoxicity with mean values of −0.30%, 6.33%, and 3.24% in HeLa, McCoy, and A549 cells, respectively (Fig. 5A, B and C). Notably, these percentages fall well below the previously established cytotoxicity threshold of 30% (79), indicating the non-cytotoxic nature of SbPh_4_ACO toward these mammalian cell lines at this concentration. This lack of cytotoxicity of SbPh_4_ACO coupled with the synergistic effect with cefoxitin signifies the potential combinatory application of this antimicrobial against MRSA.

At 25 µg/mL, SbPh_4_ACO showed a mean cytotoxicity of 22.37% in HeLa cells and higher cytotoxicity in McCoy and A549 cells which exceeded the threshold of 30%. When used at concentrations above 50 µg/mL, the cytotoxicity of SbPh_4_ACO exceeded 30% in all three cell lines (Fig. 5; Fig. S1). It is noteworthy that the levels of SbPh_4_ACO cytotoxicity varied in different cell lines which highlights the importance of including several lines in testing.

The strategy of using non-antibiotic drugs as adjuvants to boost the effectiveness of existing antibiotics presents a unique method to address the crisis of antibiotic resistance and has gained interest over the past few years (13, 80–83). Several metal-based compounds have been studied for their synergistic effects with other antibiotics against resistant bacterial strains including another group V metalloid, bismuth, which potentiates tigecycline and meropenem against resistant strains of *E. coli* and *Helicobacter pylori* (13, 82, 83). Therefore, the observed antibacterial potential of organoantimony antimicrobial compound SbPh_4_ACO, especially in combination with other antibiotics at concentrations showing no (below the 30% threshold) cytotoxic effects, paves new ways in the development and future applications of metal-based antimicrobial compounds to combat the emerging bacterial resistance.

The cytotoxicity of SbPh_4_ACO, although limiting its application as an antimicrobial, may generate novel opportunities in anticancer treatments. In agreement, previous reports have shown that antimony-based compounds such as C_12_H_20_N_2_O_4_SbCl and C_14_H_24_N_2_O_4_SbCl have anticancer properties, exhibiting cytotoxicity in the cancer cell line A549 (84). Future work will address both the antimicrobial and cytotoxic effects of these compounds in an *in vivo* animal model.

### Conclusions

The present study confirmed the antibacterial activity of three tetraphenyl cyanoximates against Gram-negative or Gram-positive bacteria, *P. aeruginosa*, *E. coli*, and *S. aureus*. Among them, SbPH_4_ACO demonstrated bactericidal activity against all three pathogens tested. Exposure to SbPH_4_ACO causes membrane damage and increases membrane permeability. The combinatory treatments with SbPH_4_ACO and two clinically used antibiotics, polymyxin B and cefoxitin against *P. aeruginosa* and *S. aureus*, respectively, showed synergistic impact. Furthermore, at the synergistic MIC concentration 12.5 µg/mL, SbPh_4_ACO showed no (below the 30% threshold) cytotoxicity against the mammalian cell lines HeLa, McCoy, and A549. The results indicate that SbPh_4_ACO holds promise as an effective treatment alone or in combination with antibiotics against resistant bacterial infections caused by important human pathogens such as *P. aeruginosa*, *E. coli*, and *S. aureus*.

## MATERIALS AND METHODS

### Media and antimicrobial compounds

Low-salt Luria-Bertani (LB) medium was used for antimicrobial susceptibility screening. For susceptibility testing and determination of minimum inhibitory concentrations for selected antimicrobial compounds, CA-MHB (Sigma-Aldrich, catalog no. 90922) was used as per CLSI standards (85).

### Bacterial strains

Three bacterial pathogens used include Shiga-toxin producing *E. coli* strain S17(O113:H4) isolated from a chick liver with septicemia (86), respiratory methicillin-resistant *S. aureus* strain NRS70 (87), and lab-adapted burn wound *P. aeruginosa* isolate PAO1 (88). All strains were maintained in 10% skim milk at −80°C. Before each experiment, the bacterial strains were inoculated onto LB agar from frozen stocks and grown overnight at 37°C from which isolated colonies were picked for precultures.

### Synthesis and characterization of Sb-based antimicrobial compound

**Fig 6:**
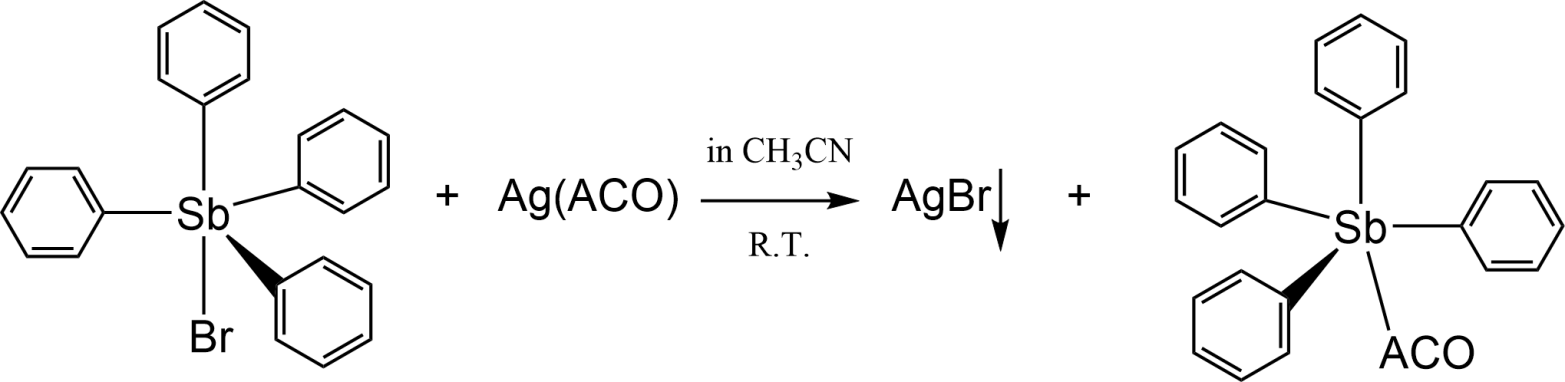
The most convenient route to target organoantimony(V) cyanoximate.

Preparation of organometallic compounds used in our investigation is shown in Fig. 6 where we used the heterogeneous metathesis reaction between the solid Silver(I) salt of the 2-oximino-2-cyano-acetamide (cyanoxime ACO) and solution of the tetraphenylantimony(V) bromide. The cyanoxime was obtained according to our previous work (35, 89), while the latter antimony source was prepared according to published procedures (90, 91). Fine powder of silver(I) bromide can’t be easily filtered, and we applied the centrifugation procedure to obtain clear and colorless solution of target SbPh_4_(ACO) over pellet of AgBr as shown in Fig. S2. The solution was carefully transferred in a small beaker and was placed in a vacuum desiccator containing a beaker with paraffin turnings to absorb the solvent (CH_3_CN) and after ~1-week colorless plates and blocks of the SbPh_4_(ACO) compound begin to form at the bottom. The compound was harvested, air-dried, and packed into a vial for storage and further characterization and studies. The composition of the SbPh_4_(ACO) was established by elemental analysis on C, H, and N contents by the combustion method with the help of Atlantic Microlab (Norcross, GA; USA). The % composition of each element in C_27_H_22_N_3_O_2_Sb is presented as theoretically calculated (experimentally detected): C – 59.81 (58.39), H – 4.09 (4.16), N – 7.75 (7.29). SbPh_4_(ACO) melts at 199.5°C and decomposes rapidly after 235°C. The vibrational spectrum of the compound in KBr comprises of the following bands (cm^−1^): ν(NH_2_) – 3,439, 3,366; ν(C≡N) – 2,222; ν(C = O, amide) – 1,679; ν(C = N, oxime) – 1,478; ν(N-O, oxime) – 1,086; and ρ(NH_2_, amide) – 1,580. The compound is well soluble in common organic solvents such as acetone, acetonitrile, alcohols, DMSO, and DMF but is not soluble in hydrocarbons and water.

Further characterization of SbPh_4_(ACO) included the ^13^C{^1^H} NMR spectroscopy and the X-ray single crystal analysis. Details of crystal and refinement data as well as the most important bands (Å) and valence angles in the structure can be viewed in Tables S1 and S2 while brief description of the structure is given in Fig. 1B; Fig. S3.

### Time-kill assays

Cultures of *P. aeruginosa* PAO1, *E. coli* S17, and *S. aureus* NRS70 grown in CA-MHB for 12 h shaking at 200 rpm, 37°C, were normalized to OD_600nm_ of 0.3 to be inoculated into 2 mL of fresh CA-MHB at 1% inoculum size. Thus, inoculated main cultures were grown under the same conditions for 5 h until the OD_600nm_ of 0.4–0.6 and then normalized to OD 0.1 (PAO1), 0.05 (S17), and 0.05 (NRS70) to achieve 1.05 × 10^7^ CFU/mL. Then, 25 µL of the normalized cells was inoculated into a 24-well clear plate carrying 500 µL CA-MHB, and this dilution provides a cell density of 5 × 10^5^ CFU/mL, recommended by CLSI for time-kill assays (92). CA-MHB is supplemented with SbPh_4_ACO in the concentrations ranging from 0.25× MIC to 4× MIC, corresponding to each strain. The control wells contained CA-MHB with no antimicrobial. The bacterial cultures were incubated at 37°C shaking at 200 rpm, and 10-µL aliquots were collected at time points 0, 1, 3, and 6 h post-SbPh_4_ACO treatment. The aliquots were serially diluted 10^−1^–10^−8^ in sterile 0.9% NaCl, and 5 µL of each serial dilution was plated on LB agar for CFU count. For data representation, a CFU value of 0 was replaced with 1 to allow use of the logarithmic scale. Each experiment was based on at least three replicates and repeated at least once. The reported CFU values represent the mean value along with standard error. The MBC value was determined as the concentration of the antimicrobial that eliminates at least 90% of the bacterial population within 6 h upon exposure, which is equivalent to 99% killing of the inoculum (3 log_10_-fold decrease in colony-forming units) at 24 h as established in references (43, 44, 93).

### Determination of minimum inhibitory concentration

Antimicrobial compounds were solubilized in DMSO at a concentration of 5 mg/mL. Their working stocks were prepared in CA-MHB at a concentration of 400 µg/mL, from which serial dilutions in the range 0.39–200 µg/mL were prepared in CA-MHB in a clear bottom 96-well plate. Individual colonies recovered from frozen stocks were inoculated into CA-MHB and grown at 37°C shaking at 200 rpm for 12 h. The bacterial cultures were normalized to an OD_600 nm_ of 0.02, which corresponded to an inoculum size of 2–8 × 10^5^ CFU/mL recommended for MIC assays (94). Then, 100 µL of thus normalized cultures was transferred into a 96-well plate containing 100 µL CA-MHB supplemented with the appropriate amount of antimicrobial compounds and incubated at 37°C shaking at 200 rpm. Untreated and cells treated with 10 µg/mL gentamicin (Sigma-Aldrich) were used as negative and positive controls, respectively. The initial and final OD_600 nm_ after 24 h incubation was measured using a Biotek Synergy Mx plate reader. The concentration of the antimicrobial compound, at which growth was not detected, was reported as the MIC.

### Checkerboard assays

The Checkerboard method was used to evaluate synergism for SbPh_4_ACO combined with polymyxin-B, meropenem, or cefoxitin against *P. aeruginosa* PAO1, *E. coli* S17, and *S. aureus* NRS70, respectively. The broth microdilution assay was performed in a 96-well plate with the final volume of 200 µL. A working stock solution of SbPh_4_ACO in CA-MHB at a concentration of 8xMIC was used to generate serial dilutions 2× MIC, 1× MIC, 0.5× MIC, 0.25× MIC, 0.125× MIC, 0.0625× MIC, and 0.03125× MIC in a clear bottom 96-well plate. Similarly, polymyxin-B, cefoxitin, and meropenem were subjected to serial dilutions to obtain their respective final concentrations ranging from 1/32 MIC to 2 MIC in CA-MHB. Aliquots of these antibiotics were placed into the 96-well plate containing SbPh_4_ACO. Bacterial cultures grown in CA-MHB at 37°C shaking at 200 rpm for 12 h were normalized to an OD_600 nm_ of 0.02. Then, 100 µL of these normalized cultures were inoculated into the 96-well plate containing 100 µL of the combined solutions of two antimicrobial compounds at the time. The plate was incubated for 24 h shaking at 200 rpm at 37°C, and the initial and final OD_600 nm_ were measured using a Biotek Synergy Mx plate reader. The MICs of each drug alone and in combination were used to calculate the FIC_I_; FIC_I_ = FIC_A_ + FI_CB_, where FIC_A_ = MIC_A+B_/MIC_A_ and FIC_B_ = MIC_B+A_/MIC_B_. MIC_A_ and MIC_A+B_ represent the MICs of drug A when used alone and MIC of drug A when used in combination with drug B, respectively. Similarly, MIC_B_ and MIC_B+A_ are MIC values of drug B alone and MIC of drug B when used in combination with drug A, respectively. Synergistic effects were observed when FIC_I_ ≤ 0.5, and antagonism was observed when FICI > 4; 4 < FICI > 0.5 indicated indifference between the drugs compared as established in references (67, 70).

### Preparation of cell samples for TEM

Bacterial cultures grown in CA-MHB for 12 h shaking at 200rpm at 37°C were normalized to OD_600nm_ of 0.3 to be inoculated into main cultures in 80 mL of CA-MHB at 1% inoculum size. These main cultures were grown for 5 h shaking at 200rpm at 37°C and were then divided into two tubes representing the treated and control samples. SbPh_4_ACO was added to the treated sample at 1× MIC concentration, and the control sample received an equivalent volume of the solvent DMSO. The treated and control samples were incubated for 1 h under the same conditions, after which the cells were collected by centrifugation at 3,836 *g*. The cells were resuspended in 2% glutaraldehyde in 0.1 M cacodylate buffer in a volume 10-times that of the cell pellet and then fixed for 2h at room temperature. After that, the samples were washed with cacodylate wash buffer (0.18 M sucrose in 0.06 M cacodylate buffer) three times for 15 min each wash to remove the fixative. Then, the cells were post-fixed with 1% osmium tetroxide solution for 1 h and subsequently dehydrated with 50%, 70%, 90%, 95%, and 100% ethanol for 15 min each. The last step was repeated two more times. Cells were then washed three times in propylene oxide for 15 min each wash, and the final pellet was embedded into Embed 812 (Electron Microscopy Sciences). Ultra-thin sections were prepared using Leica EM UC6 ultramicrotome equipped with a DiATOME diamond knife and contrasted with 2.5% aqueous uranyl acetate and 3% lead citrate Reynold’s stain. The sections were imaged using a JEOL JEM-2100 Transmission Electron Microscope (JEOL, Tokyo, Japan) at the OSU Microscopy Facility.

### Inner and outer membrane permeability assays

The permeability of outer and inner membranes of bacterial cells was assessed using fluorescent probes N-phenyl-1-naphthylamine (NPN) and propidium iodide (PI), respectively, as previously described, with modifications (52). Briefly, bacterial cultures grown in CA-MHB for 12 h shaking at 200 rpm at 37°C were normalized to OD_600 nm_ 0.3 and used to inoculate three 5 mL of CA-MHB at a 1% inoculum size. These cultures were grown for 5 h under the same conditions and split into two tubes: one treated with 0.5× MIC concentration of SbPh_4_ACO and the other (control) with an equal volume of DMSO for 1 h. After 1 h of treatment, cells were collected by centrifugation at 2,012 *g*, washed with 5 mM HEPES (pH 7.2) buffer supplemented with 5 mM glucose, and normalized to OD_600 nm_ 0.5. Then, 100 µL of thus normalized cells was added to a black 96-well plate, to which NPN and PI were added to a final concentration of 10 µM and 5 µM, respectively. As a positive control, to a subset of DMSO-control cells, polymyxin B was added to a final concentration of 5 µg/mL. Buffer with NPN/ PI alone served as the blank controls. The final volume of each well was 200 µL. After mixing the contents, the OD_600 nm_ and fluorescence at 350 nm (excitation)/420 nm (emission) for NPN and 535 nm (excitation) /617 nm (emission) for PI were monitored for 30 min every 5 min by using a Biotek Synergy Mx plate reader. Each experiment was based on at least three biological replicates and was repeated at least three times.

### Cytotoxicity to mammalian cells

To determine the cytotoxicity of the SbPh_4_ACO compound toward mammalian cells, each cell line (HeLa, McCoy, or A549) (ATCC, Manassas, VA) was grown in the appropriate cell culture media (Eagle’s Minimum Essential Medium + 10% fetal bovine serum (FBS) for HeLa and McCoy, F-12K medium +10% FBS for A549) and incubated with cell culture media alone or with each compound diluted to a range of concentrations from 12.5 to 200 µg/mL for 24 h at 37 °C, 5% CO_2_. The Cyquant LDH Cytotoxicity Assay Kit (Thermo Fisher) protocol was followed according to the manufacturer’s instructions to determine the percentage cytotoxicity of the compound for each cell line following the incubation period.

### Statistical analysis and graphics

Two-tailed paired Student’s *t*-test was performed on GraphPad Prism version 10.0.0 for Windows (GraphPad Software, Boston, Massachusetts, USA, www.graphpad.com), to determine statistical significance. The graphics were created in BioRender (BioRender.com).

## SUPPLEMENTAL MATERIAL

Supplemental figures and tables spectrum.04234-23-s0001.pdf Fig. S1-S3; Tables S1 and S2.

ASM does not own the copyrights to Supplemental Material that may be linked to, or accessed through, an article. The authors have granted ASM a non-exclusive, world-wide license to publish the Supplemental Material files. Please contact the corresponding author directly for reuse.
