# Supplementary material for: Novel antimony-based antimicrobial drug targets membranes of Gram-positive and Gram-negative bacterial pathogens
Source: Microbiol Spectr. 2024 Apr 23;12(6):e04234-23. doi: 10.1128/spectrum.04234-23 (PMC11237720; doi:10.1128/spectrum.04234-23)
Supplement: Supplemental figures and tables — Fig. S1-S3; Tables S1 and S2. [file spectrum.04234-23-s0001.pdf]

SUPPLEMENTARY INFORMATION

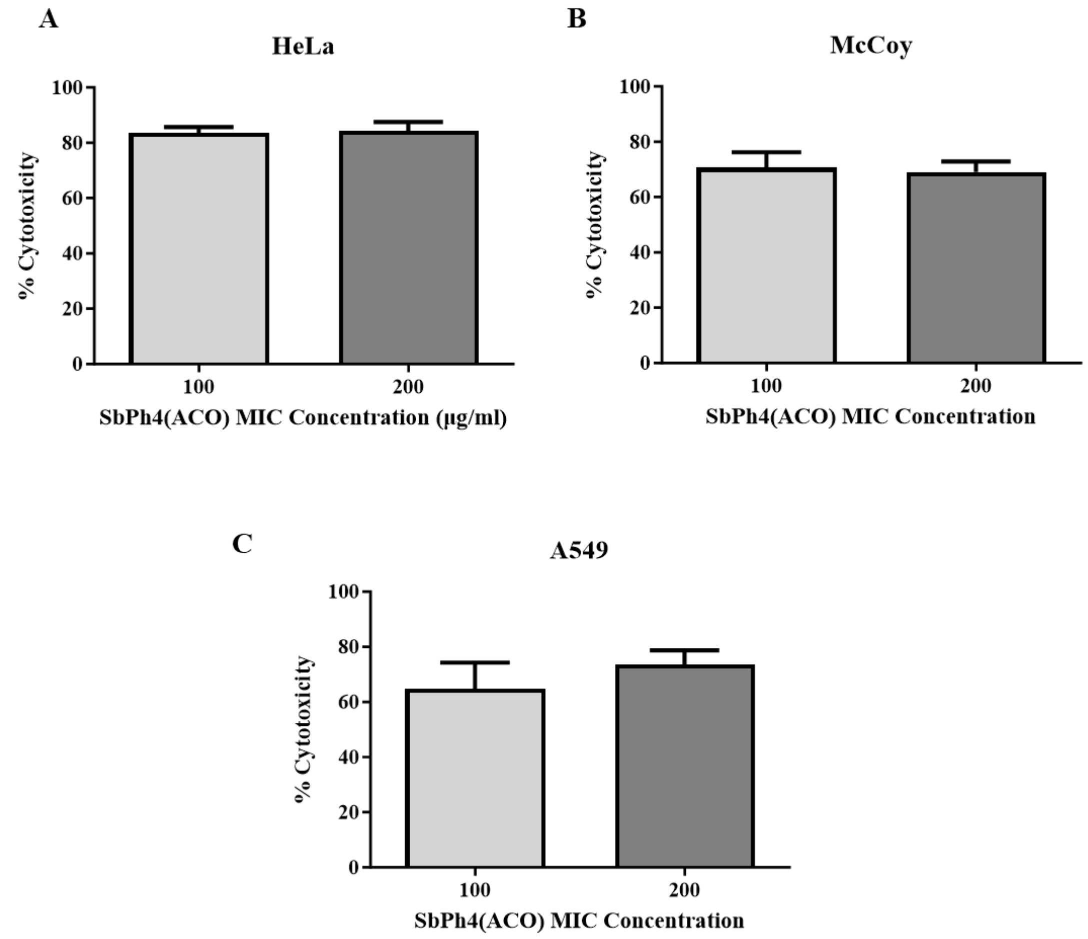

Figure S1. Cytotoxicity of experimental SbPh$_4$ACO compound. Cytotoxicity was performed using HeLa (A.), McCoy (B.), and A549 (C.) cells incubated (24 h, 37°C, 5% CO_2) in cell culture media along with SbPh$_4$ACO at concentrations 100 and 200 µg/ml concentrations. Percent cytotoxicity was determined per the manufacturer's instructions. Each experiment was conducted in triplicate wells with each cell line, and the data shown are the means ± SEM of the two independent experiments with each cell line. All compounds exhibited cytotoxicity (<60%) at concentrations above 100 µg/ml in all cell lines.

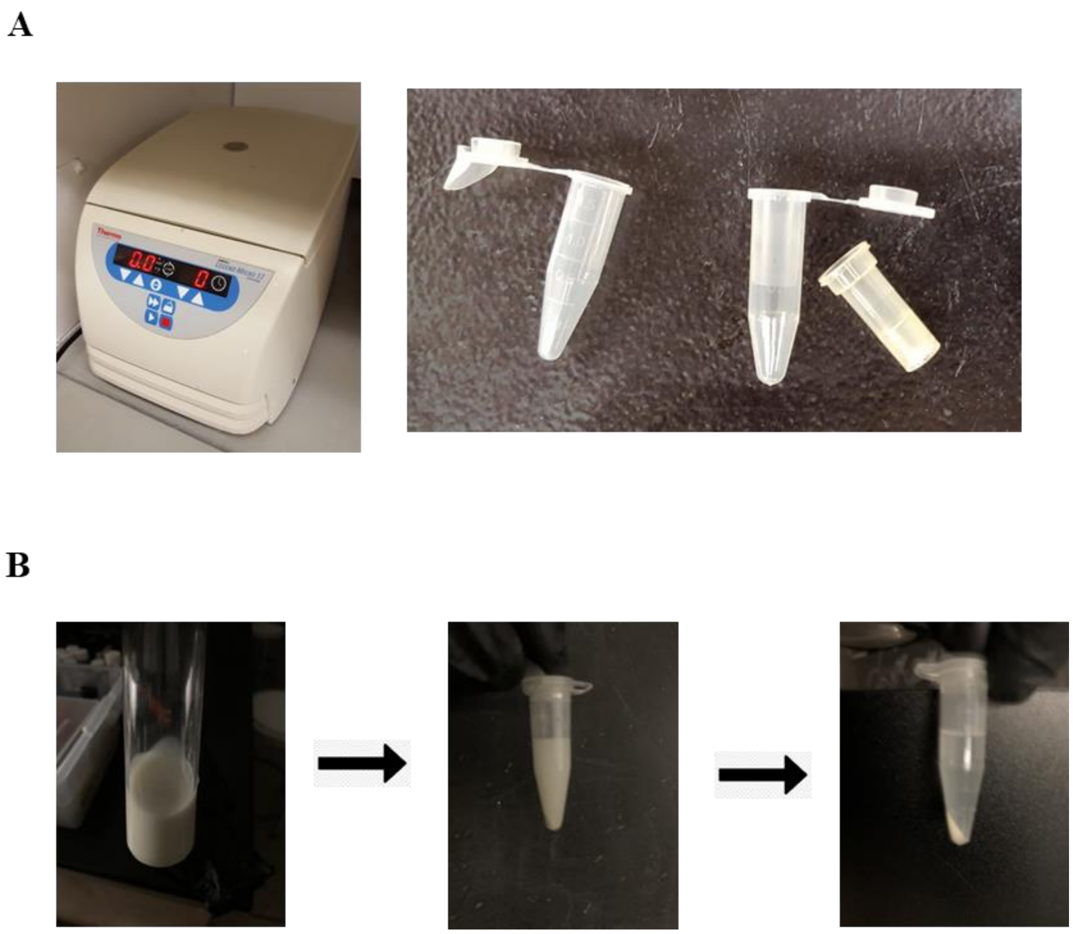

Figure S2. Instrumentation and accessories used for isolation of organoantimony(V) cyanoximates from Ag(I)Br. A Thermo Scientific centrifuge (left) with filtered nylon Eppendorf tubes (right). **B.** Progression of separation of the reaction mixture using centrifugation of a very fine precipitate of AgBr from $Sb(Ph)_4(ACO)$ solution in CH_3CN (left), transferred into an Eppendorf tube (middle), and after centrifugation showing the pellet of silver(I) bromide (right).

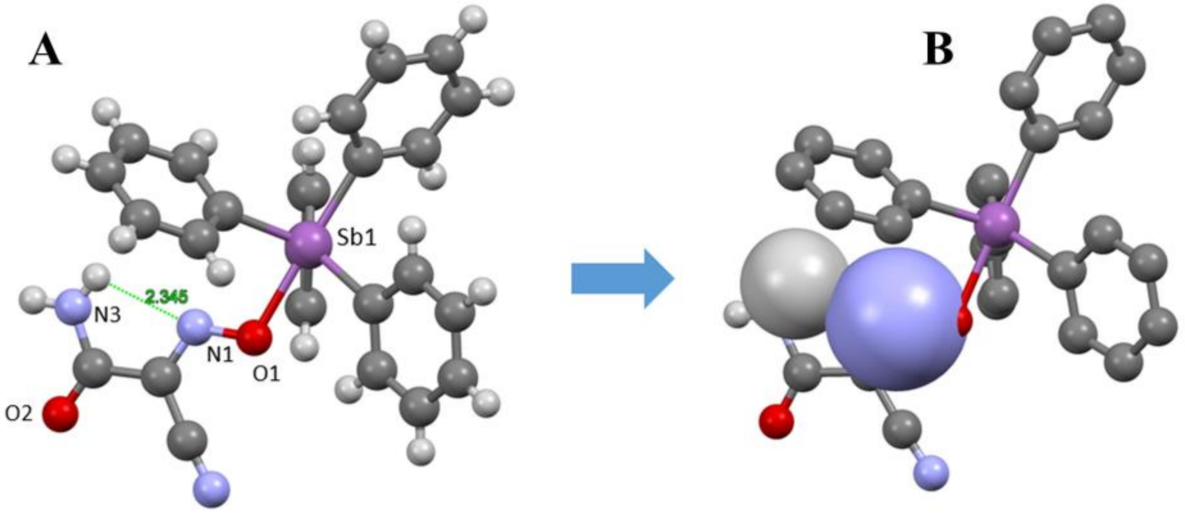

Figure S3. Two least obstructed views of $Sb(Ph)_4(ACO)$ showing structure stabilization through intramolecular H-bonding of H1a and N1 (A) along with the space filling representation of involved atoms emphasizing interactions (B).

Table S1. Crystal data and details of refinement in the structure of $Sb(Ph)_4(ACO)$.

Parameter	$Sb(Ph)_4(ACO)$	
Emperical formula	$C_{27}H_{22}N_3O_2Sb$	
F.W., g mol^{-1}	542.22	
Color	Colorless	
Crystal size, mm	0.188 mm x 0.127 mm x 0.104 mm	
Temperature, K	120(2)	
Crystal system	monoclinic	
Space group, #	P 1 21/c 1	
Unit cell, Å,°	a = 14.8336(8)	α = 90
	b = 9.9060(6)	β = 112.7130(10)
	c = 17.3977(10)	γ = 90
Unit Cell volume, $Å^3$	2358.2(2)	
Z	4	
Density (calc.) Mg m^{-3}	1.527 g/cm^3	
Absorp. Coeff., mm^{-1}	1.199	
F(000)	1088	
Θ range, °	1.49 to 33.02°	
Index ranges	-22 ≤ h ≤ 22	
	-14 ≤ k ≤ 15	
	-26 ≤ l ≤ 26	
**Structure solution**		
Reflections collected	38365	
Indpendent reflections	8465 [R(int) = 0.0278]	
Completeness to Θ, (%)	33.02° (95.0)	
Aborption correction	Multi-scan	
T_{max} and T_{min}	0.880 and 0.683	
**Refinement method**		
Data/restraints/parameters	8465 / 0 / 386	
Goodness-of-fit on F^2	1.172	
Final R indices [I > 2σ(I)]	R1 = 0.0288	
	wR2 = 0.0636	
R indices (all data)	R1 = 0.0391	
	wR2 = 0.0726	
Largest peak/hole, e $Å^{-3}$	2.183 and -0.806	
Extintion coefficient	n/a	
Structure volume, $Å^3$ (%)	1507.5 (63.92)	

Table S2. Selected bond lengths (Å) and angles (°) of the cyanoxime and metal complex center in $Sb(Ph)_4(ACO)$.

Bond length (Å)	Valence angle (°)
Cyanoxime:	
C1 - C2 = 1.441(3)	O1 - N1 - C1 = 116.81(17)
N2 - C2 = 1.144(3)	N1 - C1 - C2 = 121.02(19)
C1 - C3 = 1.498(3)	N1 - C1 - C3 = 119.22(19)
C3 - O2 = 1.227(3)	C1 - C2 - N2 = 175.9(3)
C3 - N3 = 1.339(3)	C1 - C3 - N3 = 114.25(19)
O1 - N1 = 1.337(2)	C1 - C3 - O2 = 121.0(2)
N1 - C1 = 1.278(3)	N3 - C3 - O2 = 124.8(2)
Metal Center:	
Sb1 - O1 = 2.2529(14)	C4 - Sb1 - O1 = 81.29(7)
	C10 - Sb1 - O1 = 176.48(6)
	C16 - Sb1 - O1 = 84.74(7)
	C22 - Sb1 - O1 = 83.43(7)
	C10 - Sb1 - C4 = 95.71(8)
	C16 - Sb1 - C4 = 118.80(8)
	C22 - Sb1 - C4 = 120.76(8)
	C16 - Sb1 - C10 = 98.35(8)
	C16 - Sb1 - C22 = 116.21(8)
	C22 - Sb1 - C10 = 96.62(8)
